# Cell Membrane-Coated Oil in Water Nano-Emulsions as Biomimetic Nanocarriers for Lipophilic Compounds Conveyance

**DOI:** 10.3390/pharmaceutics13071069

**Published:** 2021-07-12

**Authors:** Martina Profeta, Concetta Di Natale, Elena Lagreca, Valentina Mollo, Paolo Antonio Netti, Raffaele Vecchione

**Affiliations:** 1 Center for Advanced Biomaterials for Health Care (CABHC), Istituto Italiano di Tecnologia, Largo Barsanti e Matteucci 53, 80125 Napoli, Italy; martina.profeta@unina.it (M.P.); concetta.dinatale@unina.it (C.D.N.); elena.lagreca@iit.it (E.L.); Valentina.mollo@iit.it (V.M.); paolo.netti@iit.it (P.A.N.); 2 Interdisciplinary Research Centre on Biomaterials (CRIB), University of Naples Federico II, P.le Tecchio 80, 80125 Naples, Italy; 3 Department of Chemical Materials and Industrial Production (DICMAPI), University of Naples Federico II, P.le Tecchio 80, 80125 Naples, Italy

**Keywords:** nanoemulsions, chitosan, cell membrane, CM-NEsoSomes, biocompatibility

## Abstract

Recently, we developed ultra-stable oil in water nano-emulsions (O/W NEs), able to carry both internal and external cargos (Somes), such as lipophilic compounds and hydrophilic coatings, respectively, that we call here NEsoSomes. O/W NEs are an excellent bioengineering tool for drug and molecules delivery, due to their ability to dissolve a large number of hydrophobic compounds and protect them from hydrolysis and degradation under biological conditions. At present, no report is available on the combination of cell membrane coatings with such nanocarriers, probably due to their typical instability feature. Since then, we have reported, for the first time, a new cell membrane (CM)-coated nanomaterial composed of membranes extracted from glioblastoma cancer cells (U87-MG) deposited on NEsoSomes, through a liquid–liquid interface method, to produce highly controllable membrane caked nano-capsules, namely CM-NEsoSomes. CM-NEsoSomes were physically characterized by dynamic light scattering (DLS) over time and their correct morphology was analyzed by confocal and transmission electron microscopy (TEM) microscopy. Moreover, CM-NEsoSomes biocompatibility was tested on the healthy model cell line, performing cell cytotoxicity and uptake assay, showing nanocarriers uptake by cells with no induced cytotoxicity.

## 1. Introduction

Nanoscale drug delivery systems (NDDS) are widely investigated to improve the efficacy and safety of drug therapy and diagnostics. Among these, oil in water nano-emulsions (O/W NEs) are an ideal system for the encapsulation of lipophilic molecules [1]. The kinetic stability of O/W NEs is usually increased via layer-by-layer strategies, and their surfaces can be decorated with specific ligands, such as proteins, polymers and cell-penetrating peptides (CPP) able to target cells or tissues [1,2,3,4,5]. However, they have some limitations, including short circulation time, immune recognition, poor tumour accumulation and penetration [3,6,7]. Recently, improving nano-carrier circulation times has gained considerable attention and, among all current proposed strategies, the most innovative one is based on biomimetic systems derived from extracted cell-membranes [8]. Cell membrane-coated nano-carriers consist of a core formed by different materials, and in a shell of cell-derived membranes; this coating strategy is known to elude the immune system, simplify in vivo imaging, improve therapeutic efficacy and increase drug accumulation in specific targeted sites, depending on the nature of the coated membrane [7,8]. Many types of cell membrane-coated nano-carriers based on external shell of membranes from red blood cells [9,10,11], white blood cells [9], cancer cells [8,12] and bacteria [13] with a synthetic core of different kinds of materials such as polymers [10], gold [11], and silica [14] are reported in the literature. The current cell membrane-coated nano-carriers are obtained by electrostatic attraction [6], sonication method [15] and by cell-membrane-templated gelation technique [16], microfluidic system [17], in situ packaging of nanomaterials using live cells [18], which offer a full coating of the particles and retaining cell surface proteins in a right-side-out manner [15]. Up to now, the only example of cell membrane-coated nano-emulsion is based on recent red blood membrane-coated perfluorocarbons (PFC) nanoformulation, which has been reported as an oxygen delivery vehicle [19]. Despite the excellent obtained results, it is well-known that perfluorinated compounds are not biodegradable and most of them show a poor solubility in ethanol or acetone, which are the most common solvents used to solubilize/disperse several compounds for therapeutic or diagnostic purposes. By contrast, vegetable oil involved in our O/W NEs, is biocompatible, biodegradable and highly miscible with ethanol and acetone, and so able to encapsulate lipophilic substances such as curcumin [5], lycopene [20] and co-enzyme Q10 [21]. Moreover, our O/W NEs coated by a layer of chitosan (Ct), namely secondary nano-emulsions (SNE), were optimized in terms of formulation and process, in order to provide a shelf life over 1 year [22] and a consequent exceptional ability to be stably coated with several materials, such as polymers, as well as inorganic materials [23,24,25].

**Scheme 1:**
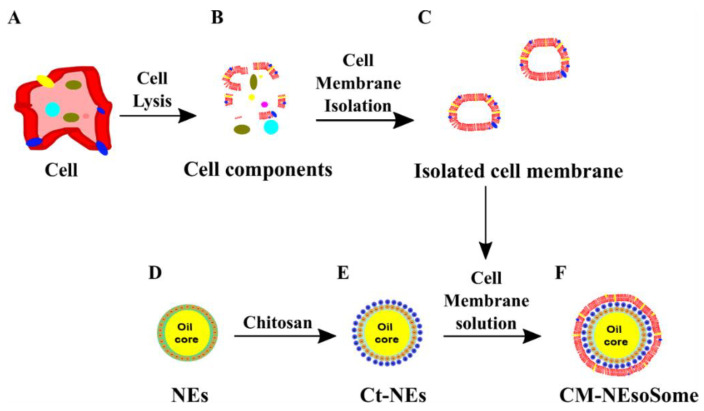
Schematic representation of CM-NEsoSome preparation: (**A**) Cell, (**B**) Cell components, (**C**) Isoleted Cell Membrane, (**D**) O/W NEs, (**E**) chitosan deposition and (**F**) Final CM-NEsoSome.

In light of these considerations, here we present, for the first time, a biocompatible and nature-based O/W NE –membrane system (CM-NEsoSomes) as a potential biomimetic nanocarrier of lypophilic compounds. Cell membranes were extracted from U87-MG cells and, after membrane purification, the layer was obtained through a liquid–liquid interface by an electrostatic interaction between negatively charged cell membrane and positively charged Ct-NE (Scheme 1). Furthermore, CM-NEsoSomes were characterized and their biocompatibility was tested on HDF, by means of cell cytotoxicity and uptake assay.

In this scenario, thanks to the Ct-NE versatility and the biomimetic feature provided by the cell membrane coating, one can envision innovative and highly performing delivery systems with increased bioavailability and metabolic stability of the carried drugs.

## 2. Materials and Methods

### 2.1. Materials

Both soybean oil (density at 20 °C of 0.922 g mL^−1^) and surfactant Lipoid E80 (egg lecithin powder 80–85% enriched with phosphatidyl choline (PC) and 7–9.5% content in phosphatidyl ethanolamine (PE)) were purchased from Lipoid GmbH (Ludwigshafen am Rhein, Germany). Millipore Milli-Q water was used for the preparation of all nano-emulsions and solutions. Fluorescein-isothiocyanate (FITC) and chitosan (Ct, LMW 90–150 kDa, DDA 84% determined via 1H-NMR) were purchased from Sigma Aldrich (Milan, Italy). Hoechst 33342, Trihydrochloride, Trihydrate (Hoechst), CellMask™ Orange plasma membrane stain (CellMask™-543) and Wheat Germ Agglutinin, Alexa Fluor™ 488 Conjugate (WGA-488) were purchased from Thermo Fisher Scientific (Waltham, MA, USA).

### 2.2. Cell Culture

Human glioma cell line (U87-MG) was purchased from ATCC^®^ and used as a cancer cells model for membrane extraction. Primary human dermal fibroblasts (HDF) were purchased from ATCC^®^ (Manassas, VA, USA) and used as a healthy tissue model, to test the biological effect of the final system.

U87-MG cells were cultured with Eagle’s minimal essential medium (EMEM) supplemented with 10% FBS, 1% of glutamine and antibiotics. Cells were sub-cultured in T150 cell culture flask for membrane extraction, in a humidified controlled atmosphere with 5% of CO_2_, at 37 °C. The medium was changed every 2–3 days.

HDF cells were cultured in EMEM supplemented with 20% Fetal Bovine Serum (FBS, Gibco), 1% of glutamine, 100 U/mL penicillin, 100 mg mL^−1^ streptomycin and 2X nonessential aminoacids. Cells were maintained in cell culture plates of 100 mm diameter in a humidified controlled atmosphere with 5% of CO_2_, at 37 °C. The medium was changed every 2–3 days.

### 2.3. Cell Membrane Isolation and Characterization

Cell membranes were isolated from the cancer cells U87-MG, according to the procedure reported by *Balasubramanian et al.* [26] with modifications.

Prior to cell detachment, cell culture medium was removed and 3 mL of a PBS solution containing 0.01% of Hoechst, 0.1% of WGA-488 and 0.1% of CellMask™-543 was added to each flask. In this way, cell nuclei, cell membrane glycoproteins and plasma cell membranes were stained, respectively. After 10 min of incubation in a humidified controlled atmosphere with 5% of CO_2_, at 37 °C, 10^9^ U87-MG cells were detached from the T150 cell culture flasks by trypsinization, and centrifuged at 500× *g* for 5 min. The resulting cell suspension was washed three times with PBS buffer by centrifuging at 500× *g* for 4 min, and then suspended in hypotonic lysing buffer at a 1:10 ratio pellet/lysis buffer. The lysis buffer was composed as follows: 20 mM Tris-HCl pH 7.5; 10 mM KCl; 2 mM MgCl_2_. The cells were disrupted by pipetting them thoroughly and spinning the solution at 3.200× *g* for 5 min. The resulting pellet was dissolved again in the hypotonic lysing buffer solution, pipetted and spun down by centrifugation at 3.200× *g* for 6 min. After that, the supernatants were collected, mixed and centrifuged at 18.000× *g* for 20 min at 4 °C. The pellet was collected, characterized and used as purified cancer cell membrane for the subsequent experiments.

The membrane extraction protocol was performed in triplicate to validate its reproducibility and the purity of the final sample. The pellets or the supernatants of each step of the purification process were observed by confocal microscope (Leica Microsystems TCS SP5 II, Wetzlar, Germany) with a 25X water immersion objective. Images were acquired with a resolution of 1024 × 1024 pixels (Figure S1).

For cell membrane deposition on nanoemulsion (see following paragraphs), WGA staining was avoided.

### 2.4. Circular Dichroism (CD)

CD spectroscopy is a well-established technique for studying the secondary structures of soluble proteins and it has a special relevance in the study of membrane proteins embedded in different environments, such as lipid vesicles, detergent micelles, oriented bilayers or nanoparticles [27,28].

CD spectra of membrane solutions before (371.1 µg) and after deposition (150 µg) were recorded using a Jasco J-1500 spectro-polarimeter (J-1500-150, Tokyo, Japan) in a 1.0 cm path-length quartz cell. CD spectra were registered at 25 °C in the far UV region from 260 to 190 nm [29]. The spectra were obtained with an average of 3 scans by subtracting them from blank samples (Figure S2) [30,31,32,33,34,35,36].

### 2.5. Bicinchoninic Acid Assay (BCA Assay) Membrane Protein Quantification

Protein quantification of cell membranes was evaluated using the Bicinchoninic Acid assay (BCA assay) (Bicinchoninic Acid Kit, Merk, Darmstadt, Germany). The assay was carried out according to the manufacturer’s instructions [37,38]. The absorbance at 562 nm was measured using an EnSpire^®^ Multimode Plate Reader (PerkinElmer Inc., Waltham, MA, USA) and the relative titration curve was reported in Figure S3.

### 2.6. Ct-NEs Preparation

The substrate for cell membrane deposition was a secondary nano-emulsion (SNE) which consists of an O/W NE, called “primary nano-emulsion”, coated with a layer of an opposite charge polyelectrolyte (chitosan, Ct). Briefly, the oil phase was prepared by dissolving 5.8 g of Lipoid E 80 in 24 mL of soybean oil at 60 °C using an immersion sonicator (Ultrasonic Processor VCX500 Sonic and Materials, Newtown, CT, USA); after, 5 mL of an ethanol solution of fluorescein-5-isothiocyanate (FITC) (0.6 *w*/*v*%) was added to the oil phase and the evaporation of the alcoholic solvent was carried out at 60 °C for 30 min. Then, the prepared oil phase was added dropwise to the water phase (Milli-Q water) and mixed again using an immersion sonicator in order to obtain a pre-emulsion. A low temperature was maintained throughout this process by using an ice-bath. Finally, this pre-emulsion was passed at 2000 bar through the high-pressure valve homogenizer (Microfluidics, M110PS, Microfluidics, Westwood, MA, USA) to greatly reduce the initial size [19]. In the end, to achieve the Ct-NE, a first layer of Ct was deposited above the O/W NEs by following a previously developed procedure [4]. Concisely, a 0.1 M acetic acid solution of Ct pH 4 (0.2 *w*/*v*%) was prepared and was quickly added, under vigorous stirring, to the O/W NEs 20 wt% oil and kept under stirring for 15 min to allow uniform chitosan deposition. The secondary NEs were re-dispersed through a high-pressure valve homogenizer at 700 bar for around 100 continuous steps, and finally re-processed after one week under the same conditions and stored at room temperature. The final concentrations of oil and Ct were 10 and 0.1 *w*/*v*%, respectively, while the pH of the final secondary NEs was 4.24.

### 2.7. CM-NEsoSome Preparation

Starting from the positive Ct-NE, the second layer of cells membranes was deposited using two syringe pumps (HARVARD APPARATUS 11 PLUS, Holliston, MA, USA) by adapting a procedure developed for multilayer system preparation [23]. Particularly, a cell membrane solution of c.a.0.3 µg was mixed 1:1 (v:v) with the Ct-NE (10 wt% oil—0.1 *w*/*v*% Ct) suspension at a flow rate of 0.4 mL/min through two micrometric capillaries interfaced at their extremities in order to form a single drop because of the high surface tension. Each drop was then collected inside a glass tube and stored at 4 °C for further analysis.

### 2.8. Physio-Chemical Characterization and Morphological Characterization of the Ct-NE and CM-NEsoSome Particle Size and ζ-Potential Measurements

Ct-NE and CM-NEsoSome were characterized by measuring size, polydispersity index (PDI) and ζ-potential values through DLS instrument (Zetasizer ZS, Nanoseries ZEN 3600, Malvern Instruments Ltd., Malvern, UK, λ = 632.8 nm) (Table S1). All the samples were diluted up to a droplet concentration of approximately 0.025 wt% by using Milli-Q water. A detecting angle of 173° was used. A default refractive index ratio (1.5900) and three runs for each measurement (1 run lasting 100 s) were used in the calculations of the particle size distribution. ζ-potential analysis was carried out by setting 30 runs for each measurement. A stability assay was performed with the same parameters after 30 days (Table S2).

### 2.9. CM-NEsoSome Characterization by Confocal and Stimulated Emission Depletion (STED) Microscopy

For STED analysis of fluorescent Ct-NEs, the samples are prepared by following a developed procedure [23]. In particular, each sample was diluted (a 1:10 dilution) with a 20 mM acetic acid buffer solution at pH 4 and was put in a FD3510 dish for 30 min to allow it to adhere to the surface of the dish. Then, three washes with a 5 wt% DABCO antifade solution were made and the sample observation was done by leaving the central part of the dish full of DABCO. DABCO antifade was needed to reduce the bleaching effects on the dyes. Samples were imaged using a Leica TCS SP5 STED-CW gated microscope (Leica-Microsystems, Mannheim, Germany). The resolution of the microscope was estimated to be <70 nm. The STED-CW beam power was 150 mW, measured at objective back focal plane and the time gate was 1.2 ns. For each different sample, 10 images were acquired and analyzed by ImageJ^®^ software (1.52r, Fiji Is Just ImageJ) (Figure S4A–D).

The same was performed for CM-NEsoSome, adding also a laser source of 543 nm to excite the Cell Mask™-543 used to monitor cell membrane shell.

### 2.10. Isolated Cell Membrane, Ct-NE and CM-NEsoSome Characterization by Cryo-TEM Microscopy

Cryo-TEM samples of isolated membranes obtained from glioblastoma multiform U87-MG cell line, Ct-NE and CM-NESoSome 0.25 wt% oil were prepared by Plunge freezing technique by using the Vitrobot Mark IV (Fei Company). Overall, 3 μL of each specimen were applied on 200 mesh quantifoil cupper grid in Vitrobot chamber and subsequently reduced in volume by blotting for 1 s with filter paper to yield a final thin film about 100–200 nm in thickness. To prevent sample evaporation, the Vitrobot was settled to 95% humidity and 4 °C with a waiting time of 60 s before plunging in liquid propane. After grid transfer in liquid nitrogen, each sample was mounted on Gatan Cryo holder, then observed by transmission electron microscope TECNAI G2-20 (Fei Company, Hillsboro, OR, USA) equipped by Gatan CCD camera 2HS, in Cryo mode. The imaging was performed in low dose mode, at 200 KV in a range of magnification from 20,000 to 50,000.

### 2.11. Cell Viability Assessment

To evaluate CM-NEsoSome cytotoxic effects, cell survival was quantified by Alamar Blue Assay. Overall, 10^4^ HDF cells per well were seeded in a 96-well plate. Furthermore, 24 h after seeding, cells were treated with cell culture medium alone, secondary NEs, cell membranes and CM-NEsoSome at three different time points and two concentrations. The tested times were 12, 24 and 48 h, while the chosen concentrations were 4% and 10% of nano-carrier.

Cell viability was evaluated by Alamar Blue assay. Briefly, after 12, 24 or 48 of incubation, samples were washed twice with PBS to remove the non-internalized compounds and then incubated for 3 h with a solution containing 10% of Alamar Blue cell viability reagent (Invitrogen) in cell culture medium. After incubation, supernatants were collected, and their fluorescence was measured at λ_ex_ = 570 nm and λ_em_ = 610 nm (Wallac Victor 1420, PerkinElmer, Waltham, MA, USA), according to the man ufacturer’s procedure. Data were reported as the percentage of viable cells normalized to non-treated cells. All experiments were performed in triplicate.

### 2.12. Confocal Microscopy for NE Uptake in HDF Cells

5 × 10^4^ HDF cells were seeded on a 24-well plate. Cells were incubated with Ct-NE, CM as is, CM-NEsoSome and cell medium alone as positive control, at a final concentration of 10% of nanoemulsion for 12, 24 and 48 h at 37 °C in 5% CO_2_. Then, samples were washed twice with PBS to remove the non-internalized compounds and fixed with paraformaldehyde 4% for 20 min. Finally, cells were incubated with DRAQ5 (Abcam) diluted 1:1000 in PBS for 10 min at room temperature for cell nuclei staining. Samples were observed by confocal multiphoton microscope (Leica TCS SP5 MP, Solms, Germany) with a 25× water immersion objective. Images were acquired with a resolution of 1024 × 1024 pixels. All experiments were performed in triplicate. 

### 2.13. Statistical Analysis

All experiments were performed in triplicate. As for DLS analysis, the statistical analysis was performed by Zeta software 7.02 (Malverninstrument Ltd., Malvern, UK), while STED assay was obtained as follows: a number of nanoparticles >100 for both Ct-NEs and CM-NEsoSome were analyzed by fitting their extracted profile by Gaussian. This approximation was performed to obtain the size value from the full width at half maximum (FWHM) [39].

Cell viability assessment was obtained by means of spectrofluorimetric assay. The measurements for each sample were performed in triplicate and results were presented as mean ± std. dev.

## 3. Results and Discussion

### 3.1. Cell Membranes Extraction and Purification

**Figure 1:**
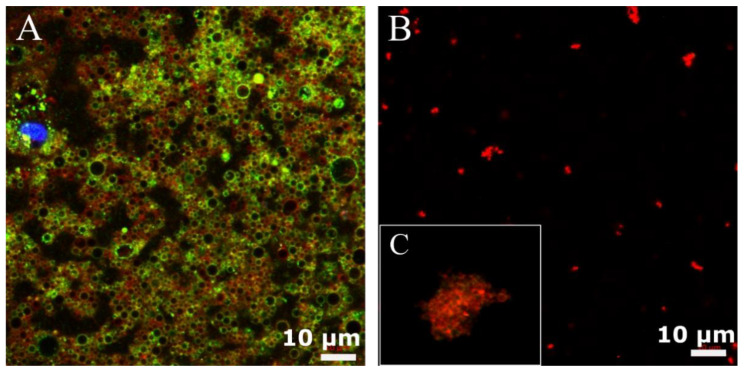
Confocal images of purified cell membrane; (**A**) cell membrane glycoproteins stained in green and plasma cell membranes stained in red, (**B**) isolated plasma cell membranes stained in red. Inset (**C**) shows a zoom-in of a single extracted membrane in figure (**B**).

Cell membranes were isolated from U87-MG according to the procedure reported by *Balasubramanian et al.* [26], with some modifications (Scheme 1A–C). The procedure consists of a gentle rupture of hypotonically swollen cells, the evacuation of most of the cell contents by repeated washing, and the isolation of cell membrane “ghosts” by a bunch of centrifugations at different speeds, avoiding the final extrusion steps. The membrane extraction protocol was performed in triplicate to validate its reproducibility and the purity of the final sample. The pellet or the supernatants of each step of the purification process were observed by confocal microscopy. Indeed, prior to membrane extraction, cell nuclei (blue) (Figure S1), cell membrane glycoproteins (green) and plasma cell membranes (red) were stained. Figure 1A shows that cell membranes integrity was preserved (red signal) as long as there was a presence of glycoproteins (green signal). Moreover, images qualitatively show a good degree of purity of the final sample. For cell membrane deposition on nano-emulsion, only the plasma cell membrane was stained (Figure 1B,C).

Moreover, in order to study the efficiency of the cell membrane extraction and purification process, together with the analysis of the correct secondary structure preserved by membrane proteins, a circular dichroism analysis was performed [27].

The results, shown in Figure S2, underline two important aspects: (i) the extraction and purification processes were performed in the correct way, and no DNA bands, usually found at 260 nm [40,41], were exposed, and (ii) membrane proteins retained their secondary structure, displaying a typical α-helix conformation with two shoulders—one negative at 205 nm and one positive at 190 nm—which are typical features of ellipticity. The same good results were obtained after the deposition process. As reported in Figure S2-blu spectrum, membrane proteins retained their ellipticity, showing the two principal α-helix related bands at 205 nm (negative peak) and 190 nm (positive peak).

### 3.2. CM-NEsoSome Preparation

As previously reported, O/W-NEs were employed as starting building blocks of CM-NEsoSome (Scheme 1). Our group has already demonstrated the ability of NEs to improve the bioavailability of lipophilic substances [5,42], making this system suitable as a potential drug delivery system [22,23], as well as diagnostic [24,43,44]. In detail, the process implemented in our laboratory allows unprecedented nanoemulsion template stability (much longer than 1 year) [23] reached thanks to the double re-dispersion of the nanoemulsion coated with chitosan (Ct-NEs). Thanks to narrow size distribution and very good stability, this system represents an ideal template for many kinds of compounds, including cell membrane, as demonstrated in this work. More in detail, by keeping good control of the mixing step, cell membrane extracted solution and Ct-NEs were injected and mixed dropwise; this strategy has previously been proven to be able to avoid aggregation mechanisms during polymer layer deposition around NEs and the solid phase.

**Figure 2:**
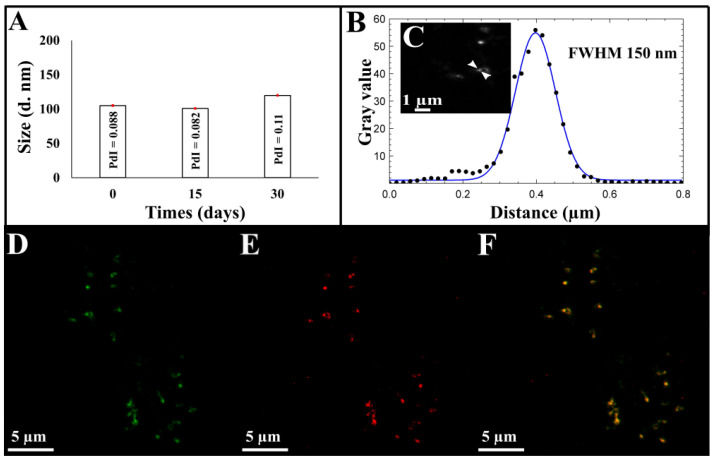
(**A**) Stability of CM-NEsoSomes over time by DLS analysis, (**B**) corresponding line profile of CM-NEsoSome intensities by STED analysis: the black dots depicted in the panel show the intensities of the corresponding pixel values in the image, blue line is the Gaussian fit of the values. Nanocarrier size was extracted from image C, (**C**) STED image of CM-NEsoSome (Scale bar 1 µm). Confocal images of CM-NEsoSomes (**D**) green channel related to FITC signal of Ct-NEs, (**E**) red channel signal of cell membrane (**F**) overlay (Scale bar 5 µm).

In the present work, we confer a new aptitude to our NE, making it a biomimetic system, thanks to the addition of a cell membrane shell able to avoid the protein corona effects and obtain a specific delivery system for targeting tumor cells [45]. Secondary NEs (Ct-NEs) were obtained from primary emulsions coated with a layer of Ct, a positive biocompatible polyelectrolyte (Scheme 1D–E). Ct deposition is fundamental for the electrostatic deposition of cell membranes. It provides a positively charged surface able to establish an attractive interaction between positive Ct-NEs droplets and negatively charged cell membranes (Scheme 1F). The cell membrane shell (371.1 ± 18.71 µg of membrane determined by Bicinchoninic Acid (BCA) assay as reported in Supplementary Materials Figure S3) was obtained by adapting a previous procedure [20,21,23,24,25,43,44,45,46]. In particular, to obtain a system with the highest degree of monodispersion (Figure 2), a single extrusion cycle was added after the liquid–liquid interface technique, in contrast with the current methodologies [47].

### 3.3. CM-NEsoSome Characterization

Size, PDI and ζ-potential of Ct-NEs and CM-NEsoSomes were evaluated by DLS analysis and the obtained results were reported in Table S1. A slight increase in terms of size and PDI was observed from Ct-NEs (96.0 ± 0.1 nm, 0.119 ± 0.02) to the final system (105.9 ± 0.66 nm, 0.088 ± 0.013), due to cell membrane shell around Ct-NEs confirming the correct deposition. Furthermore, the ζ-potential decreased in absolute value, from +38.0 ± 1.2 mV for Ct-NEs to +31.8 ± 0.88 mV for CM-NEsoSomes, stating even more the formation of the membrane shell on Ct-NEs (Table S1). CM-NEsoSomes dimensional stability over time was also evaluated by DLS analysis for 30 days (Figure 2). As reported in Table S2 and Figure S3D, the CM-NEsoSome stability was confirmed by a constant size; moreover, PDI values remained below the conventional stability rank of 0.1.

A deepest morphological analysis of our systems was performed by STED and confocal microscopy. Nanocarrier sizes were calculated as full width at half maximum (FHWM), as detailed in SI. STED results revealed, in agreement with the DLS measurements, a size value of 120 nm for the Ct-NEs and 150 nm for the final system (Figure S4A–D and Figure 2B,C) underling, as our novel deposition approach is able to maintain the correct Ct-NE and membrane protein structure (Figure S2—pink spectrum).

The efficacy of our system was also confirmed by confocal microscopy, in which, as shown in Figure 2F, a perfect match between the green color (related to FITC inside Ct-NEs) (Figure 2D) and the red one (Cell Mask™-543 colored cell membrane, Figure 2E) was revealed.

**Figure 3:**
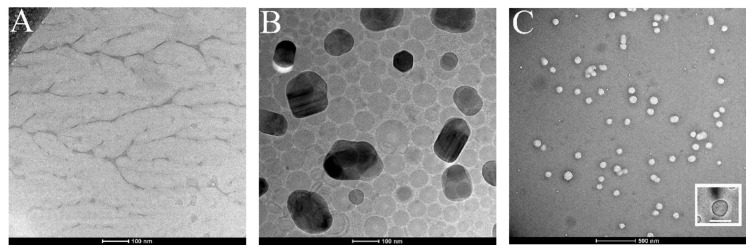
Cryo-TEM images of (**A**) purified cell membrane, (**B**) Ct-NE and (**C**) CM-NESoSome. Inset in panel **C** shows a single CM-NESoSome at higher magnification. Scale bar 100 nm.

In addition, a TEM analysis was performed. In particular, samples were imaged by using the Cyo-TEM technique in order to preserve their structure and integrity. Figure 3A,B show the isolated membranes and the Ct-NE morphology, respectively, and were used as positive control. Figure 3C shows CM-NESoSome, which appears shape-rounded and homogenously dispersed, in agreement with the DLS data. Moreover, the inset in Figure 3C clearly shows the presence of a layer on the nanoemulsion structure, further proving cell membrane deposition on the nanocarrier template, in accordance with the data reported in the confocal analyses.

### 3.4. CM-NEsoSome Biocompatibility Assessment

**Figure 4:**
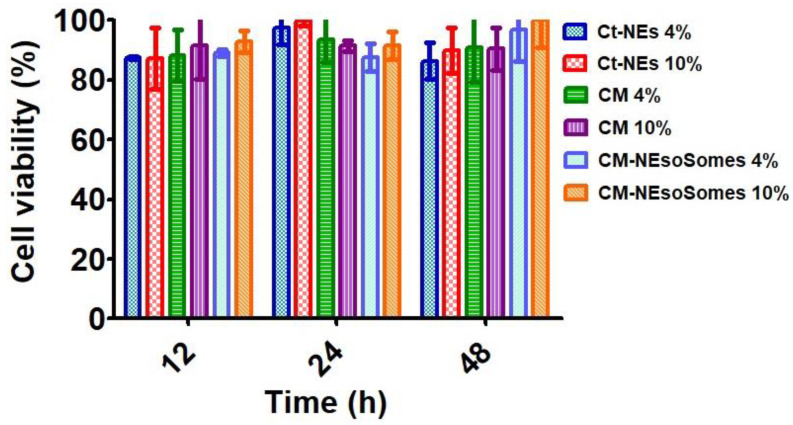
Cell Viability Assessment obtained by Alamar Blue Assay.

To verify nanocarrier biocompatibility, HDF (human dermal fibroblast) was chosen as a model line of healthy cells. HDF was incubated with the CM-NEsoSomes at a final nanoemulsion concentration of 4 and 10% for 12, 24 and 48 h. Moreover, the CM as is and the Ct-NE biocompatibility were also tested, at the same nanocarrier concentrations and incubation times, to further verify the biosafety of the nanocarrier components. Finally, cells were treated with cell medium alone as a positive control. After incubation, a quantitative evaluation of cell viability (normalized to positive control, which is set to 100%) was obtained by Alamar Blue Assay (Figure 4). Data show that all the tested compounds do not affect cell viability considerably. The latter outcomes were obtained for all contact times and all concentrations.

**Figure 5:**
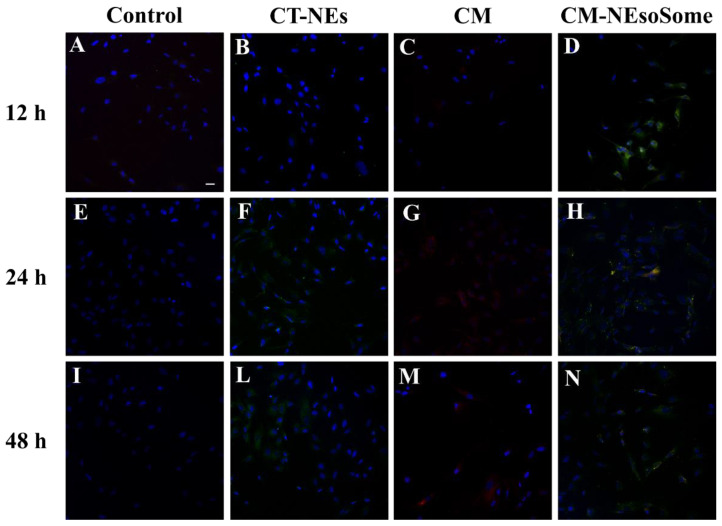
Confocal images of HDF uptake of cell medium alone (**A**,**E**,**I**), CT-NEs (**B**,**F**,**L**), CM as is (**C**,**G**,**M**) and CM-NEsoSomes (**D**,**H**,**N**) after 12, 24 and 48 h of incubation. Scale bar 30 μm.

In addition, the nanocarrier uptake by HDF cells was confirmed by confocal microscopy. Cells were treated with cell culture medium alone (Figure 5A,E,I), Ct-NE (Figure 5B,F,L), CM as is (Figure 5C,G,M), and CM-NEsoSomes (Figure 5D,H,N) for 12, 24 and 48 h, at a final NE concentration of 10% wt. The FITC-loaded Ct-NE core is represented with a green signal, the CellMask™-543-tagged membrane is visualized using a red color, while yellow hotspots show the merge of the two fluorophores in the same pixel. From a qualitative evaluation of the images, Ct-NE treated cells (Figure 5B,F,L) exhibit a spotted green signal emerging from the FITC-loaded NE that increases, increasing the incubation time. A very low signal comes from the CM as is treated cells, showing a very low uptake by cells which is probably due to the negative ζ-potential of the CM as-is (−18.5 ± 4.50 mV; Figure S5). The latter findings are in agreement with the literature; indeed, it is well known that the presence of positively charged groups on the nanoparticles surface can enhance the in vitro cellular uptake, compared to negatively charged nanoparticles [48]. Finally, the yellow spotted signal in Figure 5D,H,N originates from the colocalization of the red and green channels, suggesting that the CM-NEsoSome integrity is preserved in the extracellular environment until internalization. These observations are consistent with those of the stability tests reported in Table S2. Moreover, even in this case, the fluorescence signal increases, increasing the incubation time, and suggesting a time-dependent uptake mechanism.

Taken together, these results show that the empty nanocarrier can be uptaken by healthy cells without inducing cytotoxicity. This is a fundamental nanocarrier requirement to be assessed before moving to the applications, which in this case can range from the delivery of lipophilic drugs and biomolecules to the conveyance of lipophilic contrast agent compounds.

## 4. Conclusions

The developed nanocarrier is an innovative cell-derived biocompatible system able to combine the biological nature of cell membranes with the excellent synthetic properties of Ct-NEs in terms of stability and high drug encapsulation efficacy. These data highlight that the proposed purification and deposition process, combined with the mechanical stability of the oil core template, could be used as a powerful tool in the field of biomimetic cell-membrane camouflaged nanocarriers. Indeed, the adopted purification and deposition strategy is quite gentle, going beyond the limits of current processes that usually lead to protein degradation and instability [49], and it allows the use of oil core-based nanocarriers, which opens to the conveyance of several lipophilic compounds, ranging from drugs and biomolecules to contrast agents compounds. Therefore, these results, even if preliminary, pave the way for the possible use of this biocompatible system for the treatment and diagnosis of several human diseases, including cancer, as well as for vaccination [11] and prevention from viral infection [50]. In detail, we think that the production of mature cell membrane delivery systems (e.g., blood CM-NPs, immune CM-NPs, and cancer CM-NPs) could improve the potential of nanocarriers increasing chemotherapeutic drug biocompatibility and decreasing the incidence of associated side effects. Moreover, we believe that in the near future, the technology of cell membrane, although it has not yet achieved full clinical implementation, will yield invaluable contributions in individual precision medicine approaches.

## Supplementary Materials

The following are available online at https://www.mdpi.com/article/10.3390/pharmaceutics13071069/s1, Figure S1: Cell nuclei (blue) contained in the first pellet of the purification process. Figure (**B**) is a zoom in of Figure (**A**). Figure S2: Overlay of CD spectra of: pink) bare membrane after extraction and purification processes (371.1 µg) and blue) cell membrane proteins deposited on Ct-NE (150 µg). Figure S3: Cell membrane quantification by BCA assay. Titration curve. Table S1: Size, PDI and ζ-potential data of Ct-NEs and CM-NEsoSome. Table S2: Size and PDI of CM-NEsoSome over time obtained by DLS analysis. Data are expressed as mean ± SD (*n* = 3). Figure S4: STED analysis of Ct-NE (A,B) and CM-NEsoSome (C,D). (A,C) STED images, (B,D) Deconvolution spectra analysis by ImageJ^®^ software extracted respectively from images (A,C). Figure S5: ζ-potential (mV) distribution of U87-MG cells.
