# Supplementary material for: Cell Membrane-Coated Oil in Water Nano-Emulsions as Biomimetic Nanocarriers for Lipophilic Compounds Conveyance
Source: Pharmaceutics. 2021 Jul 12;13(7):1069. doi: 10.3390/pharmaceutics13071069 (PMC8309122; doi:10.3390/pharmaceutics13071069)
Supplement: Supplementary file 1 [file pharmaceutics-13-01069-s001.zip › pharmaceutics-1272923-SI.pdf]

pharmaceutics

MDPI

Supplementary Materials: Cell Membrane-Coated Oil in Water Nano-Emulsions As Biomimetic Nanocarriers for Lipophilic Compounds Conveyance

Martina Profeta, Concetta Di Natale, Elena Lagreca, Valentina Mollo, Paolo Antonio Netti and Raffaele Vecchione

Supplementary information

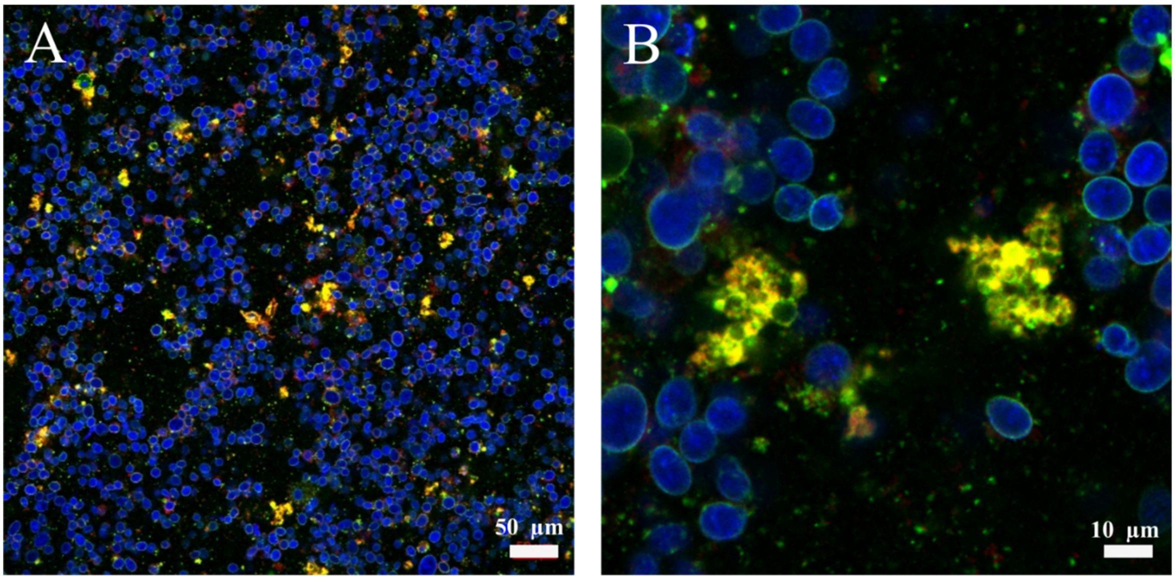

Figure S1. Cell nuclei (blue) contained in the first pellet of the purification process. Figure (**B**) is a zoom in of Figure (**A**).

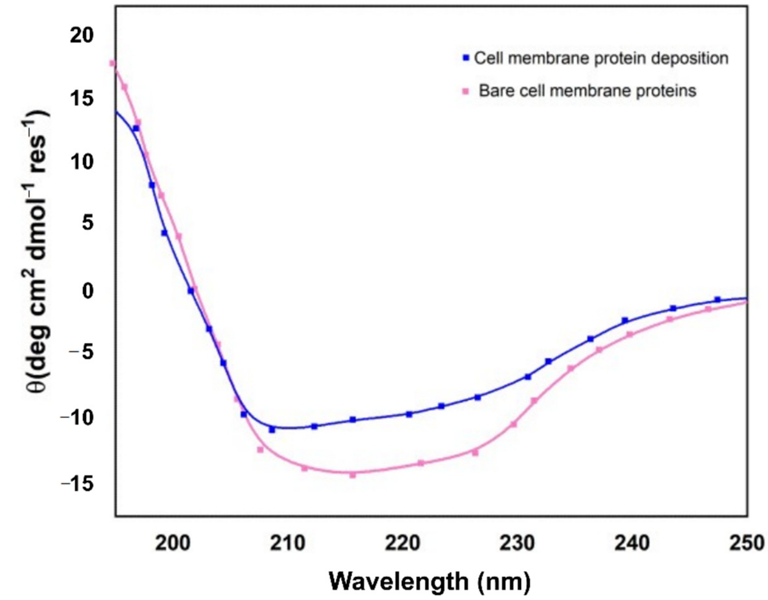

Figure S2. Overlay of CD spectra of: pink) bare membrane after extraction and purification processes (371.1 µg) and blue) cell membrane proteins deposited on Ct-NE (150µg).

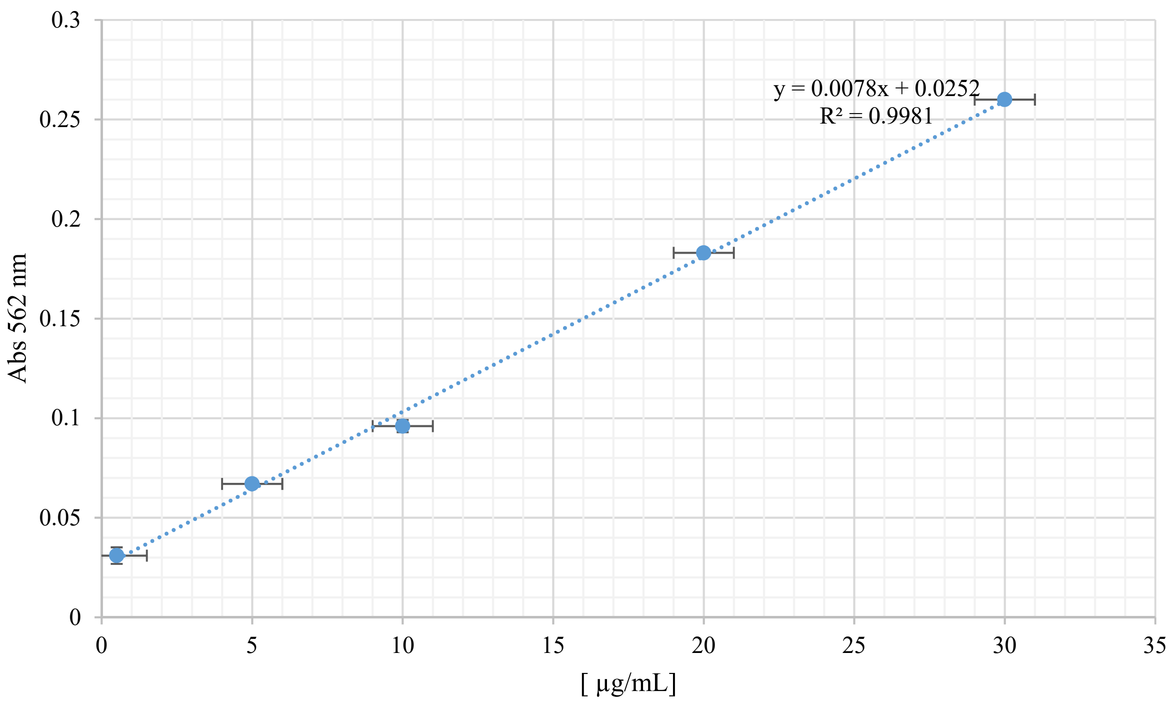

Figure S3. Cell membrane quantification by BCA assay. Titration curve.

Table S1. Size, PDI and ζ-potential data of Ct-NEs and CM-NEsoSome.

Samples	Size /d. nm	PDI	ζ-potential / mV
Ct-NEs	96.0 ± 0.1	0.119 ± 0.02	+38.0 ± 1.2
CM-NEsoSome	105.9 ± 0.66	0.088 ± 0.013	+31.8 ± 0.88

Table S2. Size and PDI of CM-NEsoSome over time obtained by DLS analysis. Data are expressed as mean ± SD (*n*=3).

Time / days	Size /d. nm	PdI
0	105.9 ± 0.66	0.088 ± 0.013
1	108.0 ± 4.71	0.086 ± 0.02
7	105.5 ± 0.089	0.089 ± 0.010
15	100.8 ± 0.55	0.082 ± 0.09
30	119.8 ± 1.02	0.118 ± 0.017

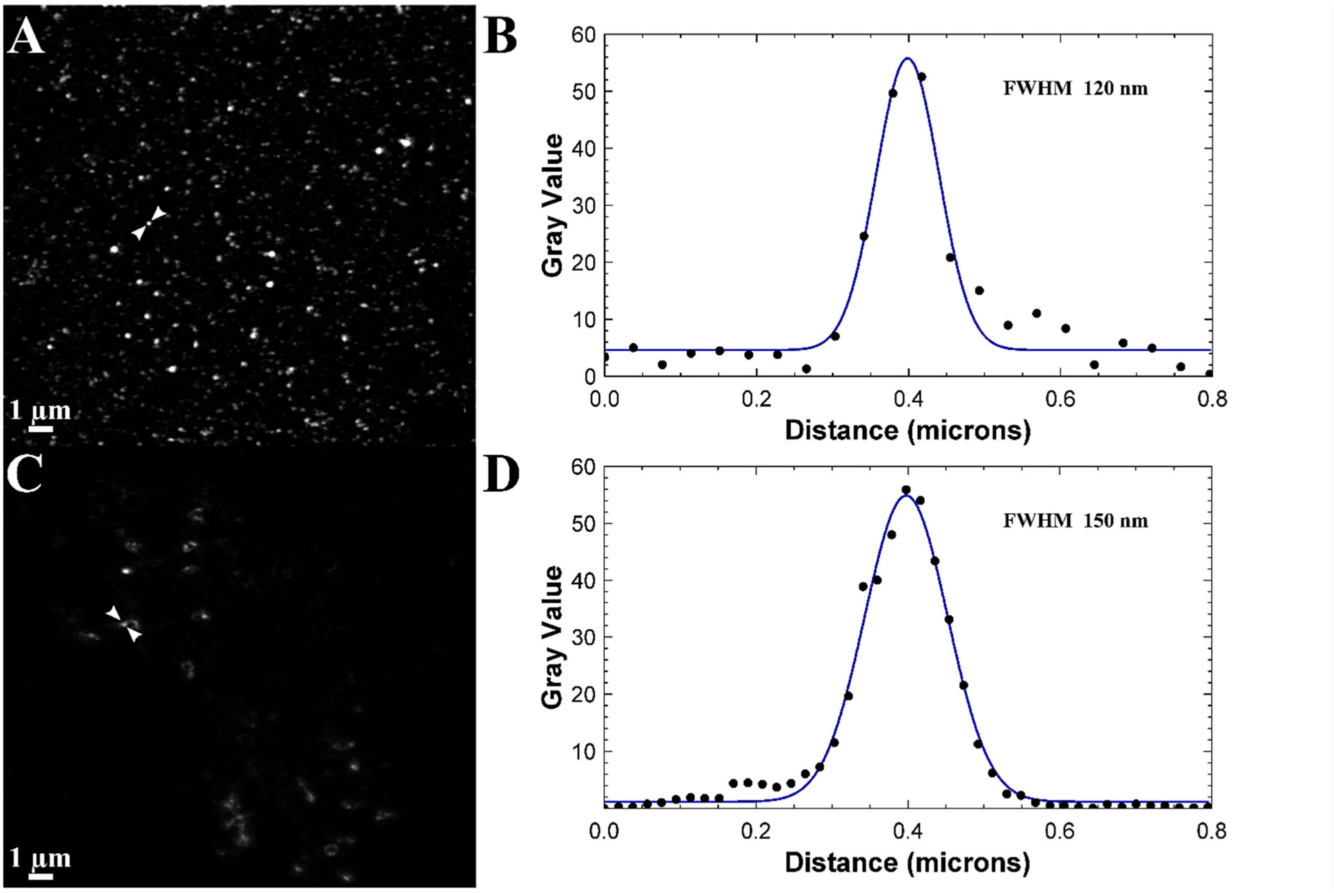

Figure S4. STED analysis of Ct-NE **(A,B)** and CM-NEsoSome **(C,D)**. **(A,C)** STED images, **(B,D)** Deconvolution spectra analysis by ImageJ® software extracted respectively from images **(A,C)**.

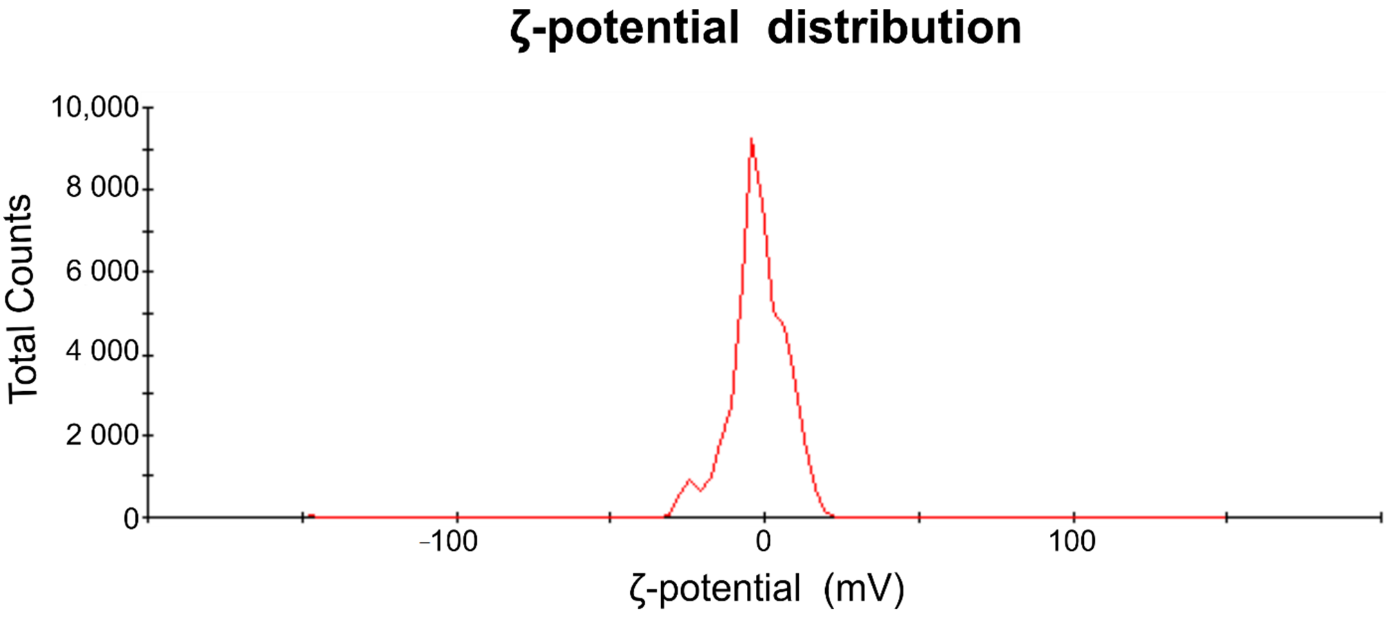

Figure S5. ζ-potential (mV) distribution of U87-MG cells.
